# Supplementary material for: Kidney function in healthcare clients in Khayelitsha, South Africa: Routine laboratory testing and results reflect distinct healthcare experiences by age for healthcare clients with and without HIV
Source: PLOS Glob Public Health. 2024 May 16;4(5):e0002526. doi: 10.1371/journal.pgph.0002526 (PMC11098392; doi:10.1371/journal.pgph.0002526)
Supplement: S1 Fig — X-axis: Age (years) of first test results. Y-axis: Density distribution. A: Age distribution at first serum creatinine results for females by HIV status. B: Age distribution at first serum creatinine results for males by HIV status. (PDF) [file pgph.0002526.s001.pdf]

**Osei-Yeboah et al. Supporting Information File S1 Fig**

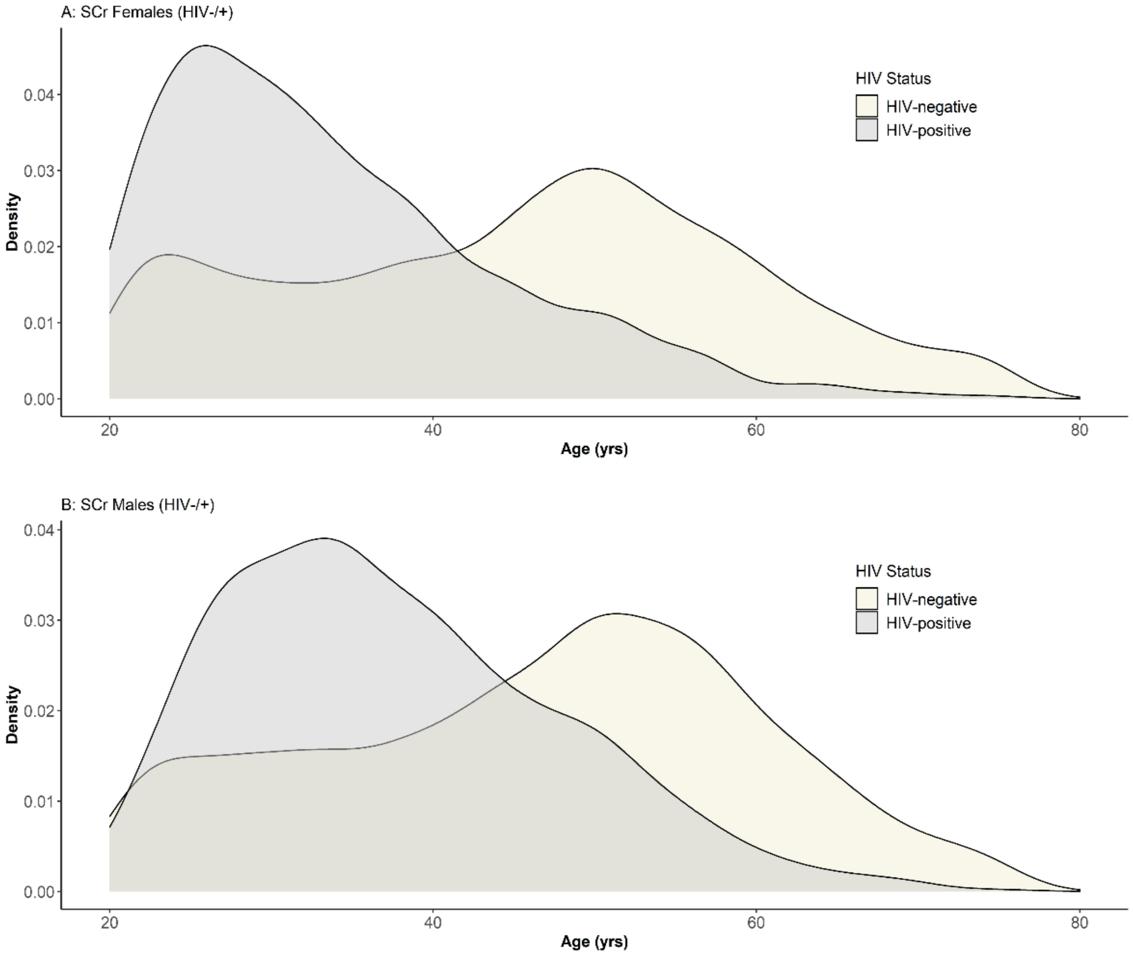


## Supporting Information

S1 Fig: **Distribution of age at first serum creatinine testing without matched estimated glomerular filtration rate for females and males per HIV status.** X-axis: Age (years) of first test results. Y-axis: Density distribution. A: Age distribution at first serum creatinine results for females by HIV status. B: Age distribution at first serum creatinine results for males by HIV status.
