# Supplementary material for: Kidney function in healthcare clients in Khayelitsha, South Africa: Routine laboratory testing and results reflect distinct healthcare experiences by age for healthcare clients with and without HIV
Source: PLOS Glob Public Health. 2024 May 16;4(5):e0002526. doi: 10.1371/journal.pgph.0002526 (PMC11098392; doi:10.1371/journal.pgph.0002526)
Supplement: S2 Fig — Data points are coloured by sex. Red: Female, Blue: Male. X-axis: Age (in years) at creatinine estimation. Y-axis: Serum creatinine results (umol/l). The dotted line shows the SCr value above which abnormal kidney function may be inferred. (PDF) [file pgph.0002526.s002.pdf]

Osei-Yeboah et al. Supporting Information File S2 Fig

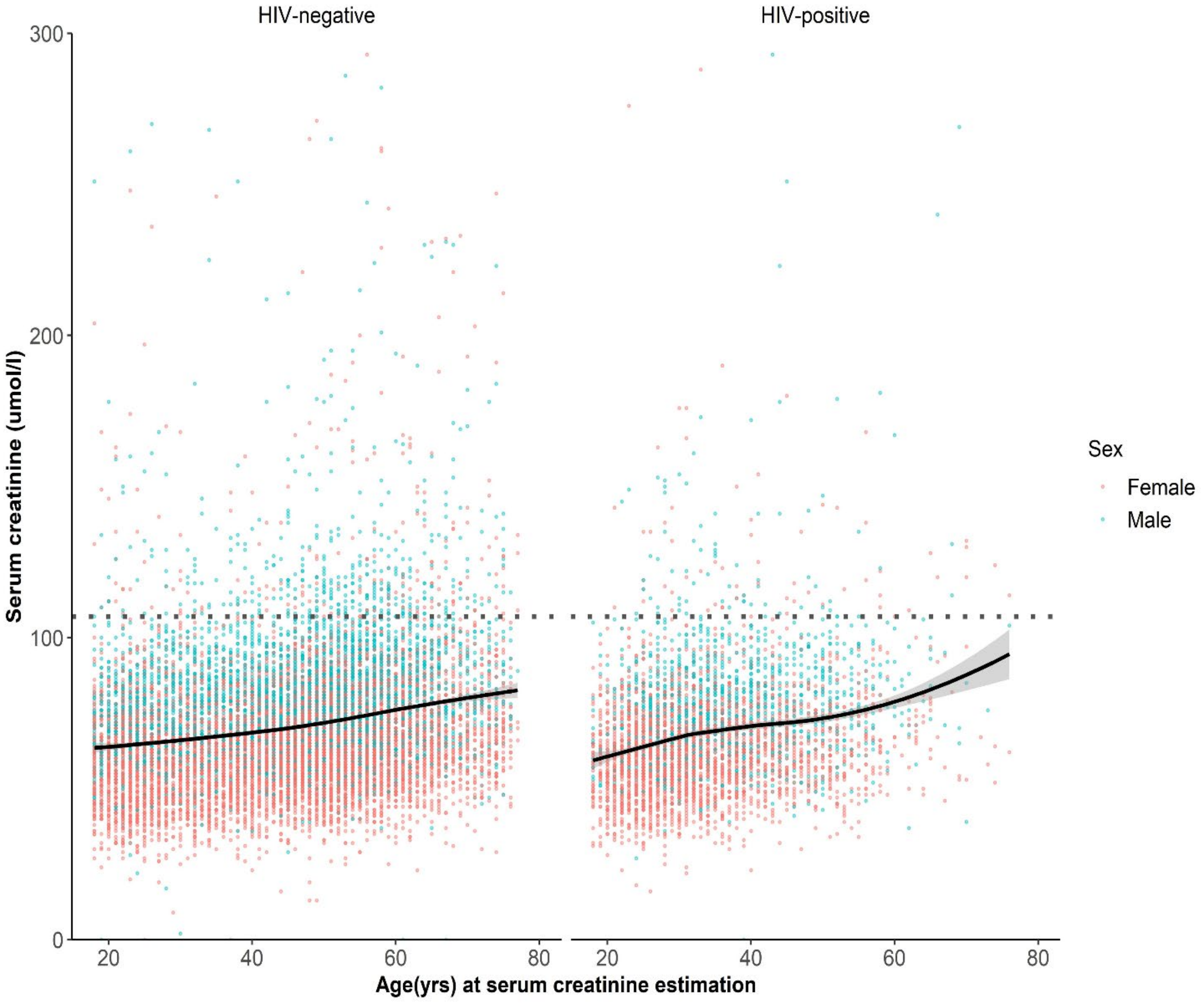

Supporting Information

S2 Fig: **Distribution of first serum creatinine without matched estimated glomerular filtration rate results per HIV status and sex.** Data points are coloured by sex. Red: Female, Blue: male. X-axis: Age (in years) at creatinine estimation. Y-axis: Serum creatinine results (umol/l). The dotted line shows the SCr value above which abnormal kidney function may be inferred.
