# Supplementary material for: Kidney function in healthcare clients in Khayelitsha, South Africa: Routine laboratory testing and results reflect distinct healthcare experiences by age for healthcare clients with and without HIV
Source: PLOS Glob Public Health. 2024 May 16;4(5):e0002526. doi: 10.1371/journal.pgph.0002526 (PMC11098392; doi:10.1371/journal.pgph.0002526)
Supplement: S1 Table — (PDF) [file pgph.0002526.s003.pdf]

Osei-Yeboah et al. Supporting Information File S1 Table

S1 Table: Characteristics of healthcare clients who received only SCr results without eGFR

		**HIV status**	
**Characteristic**	**Overall**, N = 17,729[1]	**HIV-negative**, N = 13,221[1]	**HIV-positive**, N = 4,508[1]
Age at SCr test	41 (29 – 53)	46 (31 – 55)	32 (26 – 40)
SCr (umol/l)	67 (56 – 80)	67 (57 – 81)	65 (55 – 77)
Sex			
Female	11,922 (67)	8,733 (66)	3,189 (71)
Male	5,793 (33)	4,476 (34)	1,317 (29)
Unknown	14	12	2
Tuberculosis	2,464 (14)	1,144 (8.7)	1,320 (29)
Hypertension	9,281 (52)	8,183 (62)	1,098 (24)
Diabetes	3,525 (20)	3,177 (24)	348 (7.7)
CKD	620 (3.5)	541 (4.1)	79 (1.8)

[1]Median (IQR); n (%)
