# Supplementary material for: Kidney function in healthcare clients in Khayelitsha, South Africa: Routine laboratory testing and results reflect distinct healthcare experiences by age for healthcare clients with and without HIV
Source: PLOS Glob Public Health. 2024 May 16;4(5):e0002526. doi: 10.1371/journal.pgph.0002526 (PMC11098392; doi:10.1371/journal.pgph.0002526)
Supplement: Attachment — Submitted filename: Response_to_reviewers_2.pdf [file pgph.0002526.s004.pdf]

Health Data Integration Group
South African National Bioinformatics Institute
University of the Western Cape
Robert Sobukwe Road
Belville, Cape Town 7535

Dear PLoS Global Public Health,

As per our data availability statement already provided to you, and already included in the manuscript, we are provided these data in anonymised format by our Provincial Department of Health ON CONDITION THAT we may not share them onward. Anyone wishing to use this dataset needs their application and reason for use reviewed by our Provincial Department of Health, and – as already provided in our statement and our manuscript – the details how to do this are provided accordingly.

This is not our choice, but is a condition of our permission to work with these data.

Sincerely,

From the manuscript:

"**Data availability statement**

These anonymised, perturbed data were provided for analysis by the Western Cape Department of Health, Provincial Health Data Centre. These are highly granular health data linked to individual health care clients in the province and no informed consent has been given for research use. For this reason, the Western Cape Department of Health does not permit open sharing but instead grants only primary use permission for the data. Re-use of this dataset requires approval from the Western Cape Department of Health (Provincial Health Data Centre), and Dr Moodley, Director: HIA, Western Cape Department of Health, South Africa can be contacted to advise on this process (email: melvin.moodley@westerncape.gov.za, Reference study ID 259-TIFFIN).
"

Professor Nicki Tiffin (Ph D, MPH, FAAS)
ntiffin@sanbi.ac.za
+27 83 4060134
