# Supplementary material for: Kidney function in healthcare clients in Khayelitsha, South Africa: Routine laboratory testing and results reflect distinct healthcare experiences by age for healthcare clients with and without HIV
Source: PLOS Glob Public Health. 2024 May 16;4(5):e0002526. doi: 10.1371/journal.pgph.0002526 (PMC11098392; doi:10.1371/journal.pgph.0002526)
Supplement: Attachment — Submitted filename: Response to reviewers_Osei-Yeboah_et_al.docx [file pgph.0002526.s005.docx]

**Response to reviewers**

**Title:**

**Kidney function in healthcare clients in Khayelitsha, South Africa: Routine laboratory testing and results reflect distinct healthcare experiences by age for healthcare clients with and without HIV.**

**Journal Requirements:**

1. Please review your reference list to ensure that it is complete and correct. If you have cited papers that have been retracted, please include the rationale for doing so in the manuscript text or remove these references and replace them with relevant current references. Any changes to the reference list should be mentioned in the rebuttal letter that accompanies your revised manuscript. If you need to cite a retracted article, indicate the article’s retracted status in the References list and also include a citation and full reference for the retraction notice.

**Response:** Thank you. We have reviewed the reference list to ensure it is complete and correct. We have not cited papers that have been retracted.

2. We have noticed that you have uploaded Supporting Information files, but you have not included a list of legends. Please add a full list of legends for your Supporting Information files after the references list.

**Response:** Thank you for prompting us to include the legends in the manuscript. We have added a list of legends for the supporting information files after the references list.

**Reviewer #1:**

This study investigated serum creatinine and estimated glomerular filtration rate (eGFR) results in individuals living with HIV (PLHIV) and the HIV-negative population accessing healthcare facilities in Khayelitsha, South Africa during 2016/2017. The analysis included stratification by sex, age, and the presence of comorbidities such as tuberculosis, hypertension, chronic kidney disease (CKD), and diabetes. The study's significance lies in its utilization of authentic data from routine kidney health testing among healthcare clients in Khayelitsha, providing valuable insights into the actual practices within facilities regarding kidney function testing.

**Response:** Thank you for taking the time to review our manuscript and for providing very useful comments and suggestions. We have revised the manuscript to reflect the suggested changes and we believe it has been improved.

## 1.1. One minor comment on the section "Age stratified analysis of kidney function with comorbidities TB, hypertension, diabetes, and CKD": the sentence "More tests were done for PLHIV than HIV-negative people in the 18-29 years category (35,698 vs 11,337); the 30-44 years category (93,505 vs 13,644); and the 45-60 years category (32,353 vs 26,791)" appears to incorrectly align the numbers within the brackets with the corresponding categories.

**Response:** Thank you for this observation. The numbers within the brackets have been aligned with the corresponding categories and the text reads *“More tests were done for PLHIV than HIV-negative individuals in the 18-29 years category (35 698 vs 11 337); the 30-44 years category (93 505 vs 13 644) and the 45-60 years category (32 353 vs 26 791)”.*

## 1.2. Regarding the presentation of p-values in Figure 3 and Figure 4, it might be clearer to represent them as “p =” rather than “p =<”

**Response:** Thank you for your suggestions. The p-values in Figure 3 and Figure 4 have been presented as “p =”.

## 1.3. The discussion is based on the results obtained from the study, providing appropriate analysis and comparison with similar studies. It would be valuable to discuss briefly the limitations of the study.

**Response:** Thank you for your comment on discussion. We have provided the limitations of the study in the last paragraph of the discussion on page 21 of the manuscript. The limitation paragraph reads as follows: *“A major limitation in this study is bias in the whole study population which is enriched for PLHIV and HIV-negative individuals who may be accessing care for various reasons such as accessing maternal care or accessing care because of ill health due to TB or chronic NCDs. The routine health data used in this study had a limited number of disease conditions ascertained by the PHDC and did not include important comorbidities like cardiovascular diseases (CVD). There are also challenges related to data linkage due to poor record keeping at facilities, which might result in situations where records cannot be linked or improperly linked together [35,36]”.*

## 1.4. While the conclusion provides a thorough description, it could benefit from being condensed for brevity and clarity. Additionally, ensuring consistency between the conclusion at the end of the article and the conclusion in the abstract would contribute to a cohesive presentation of the study.

**Response:** Thank you for your suggestion. We have revised the conclusion for brevity and clarity. Thank you very much for this suggestion. We have revised the conclusion in the abstract to align with the conclusion at the end of the manuscript.

**Reviewer #2:**

***General comment***

This is a well written manuscript, and the authors must be commended for putting together such a thorough manuscript!

**Response:** Thank you very much for taking the time to review our manuscript. We appreciate your very helpful comments and suggestions. We have revised the manuscript to incorporate your suggestions to improve our manuscript.

***Specific comments***

## 1.5. PHDC disease episode definition: “Microbiology culture-Toxocara canis” – This seems an unlikely target for the diagnosis of tuberculosis. Did the authors mean Mycobacterium tuberculosis?

**Response:** Thank you for the question. We have corrected the disease episode definition for tuberculosis (TB) to mycobacterium tuberculosis (MTb) to reflect use of the microbiology culture test for the diagnosis of mycobacterium tuberculosis. We apologise for our oversight of this obvious error, thank you for flagging it for us.

## 1.6. “High confidence definition of hypertension includes dispensed hydrochlorothiazide.” – Is this the only antihypertensive in use in South Africa or perhaps the most common? Would ascertainment have been improved by adding other commonly used antihypertensives?

**Response:** We believe hydrochlorothiazide is the most common antihypertensive recommended for use in South Africa by the public health sector that is reliably indicative of the patient being diagnosed with hypertension in the absence of availability clinical observational data such as repeated blood pressure readings. During episode development the PHDC algorithm is piloted on a small sample of the population and any issue is addressed before finally used for population-wide episode definitions, but these episode definitions are under continuous review and refinement to provide a more accurate ascertainment of conditions. Whilst including more types of evidence would improve the reliability of this episode, this is something that will be undertaken by the PHDC, and is not a process that we are able to undertake ourselves.

## 1.7. Data analysis: “The counts and proportions of the healthcare clients receiving eGFR results, and the ascertained comorbidities per 18-29 years, 30-44 years, 45-60 years, and over 60 years age groups were described” – Is there any reason for the choice of these specific age bands?

**Response:** Thank you for this question. Considering the mix of individuals accessing healthcare, we chose these age bands to enable us clearly to present our results to reflect the younger population, middle population, the ageing population, and the older population. We believe these age bands would clearly show the comorbidities burden in the different strata as observed, and would also help to estimate the effects of changing ART availability, since the advent of HIV, in the South African population age strata.

## 1.8. Discussion: This manuscript could have benefited from a paragraph dedicated to the strengths and weaknesses of this study. It is unclear to me whether all the PLWH were tested at ART initiation or at random points during their treatment course. It also seems the authors assumed the measurements were at random points. To address this question, it might be helpful to add to the methods section the monitoring algorithm according to the national ART guidelines for PLWH. Currently, the first line in the discussion provides a new guideline, but this new information is best placed in the methods section. It would also be helpful to discuss the question of incident HIV among older people in South Africa, and perhaps the challenge of delayed presentation among these older people (<https://pubmed.ncbi.nlm.nih.gov/35218732/>).

**Response:** Thank you for your suggestions regarding the inclusion of the strengths and weaknesses of the study. We have now highlighted the study limitations in the last paragraph of the discussion on page 21 of the manuscript. The limitation paragraph reads as follows: *“A major limitation in this study is bias in the whole study population which is enriched for PLHIV and HIV-negative individuals who may be accessing care for various reasons such as accessing maternal care or accessing care because of ill health due to TB or chronic NCDs. The routine health data used in this study had a limited number of disease conditions ascertained by the PHDC and did not include important comorbidities like cardiovascular diseases (CVD). There are also challenges related to data linkage due to poor record keeping at facilities, which might result in situations where records cannot be linked or improperly linked together [35,36]”.*

**Response:** The value of our study was presented in the conclusion section of the study on page 22 of the manuscript and reads *“The value of this study is that we have used real data about routine kidney health testing from healthcare clients in Khayelitsha, which offer insights into what is actually happening in facilities with respect to kidney function testing. This is useful for evaluating the implementation of treatment guidelines and healthcare delivery policies and service utilisation for PLHIV”.*

**Response:** The ART guideline in South Africa has evolved over the years to the current version and previous versions had recommendations for baseline laboratory evaluation and renal function assessment for adults and adolescents, pregnant women, and children. As we have indicated, the current ART guideline recommends testing of all individuals receiving ART and continuous monitoring of their kidney function even though availability of test results is not a requirement for ART initiation. Our analysis did not assess whether all PLWH were tested at ART initiation. However, the multiple test results reported for individuals indicated to us that testing was done in line with the existing guidelines. The current (updated) guidelines recommend that serum creatinine and eGFR should be assessed at 3 months and 10 months on ART (align with viral load monitoring schedule) and repeat every 12 months. Previous guidelines recommended assessment at month 6 on ART.

**Response:** We have moved the recommendation for kidney function test for PLWH in the ART guideline to the methods section.

**Response:** Thank you for your suggestion to include incident HIV among older people in South Africa in the discussion. In as much as this would be a very important addition to the discussion, we believe it fits well in another study that is under preparation for publication with a scope of assessing the relationship between HIV infection/unsuppressed viral load and ART initiation/time on ART, demographic characteristics, and occurrence of comorbidities. In this study, we analysed administratively collected routine health data for healthcare clients living with HIV who accessed care in public facilities in Khayelitsha, Cape Town. We found that in addition to ageing, the key predictors of the occurrence of HIV-related comorbidities such as TB and CKD are the presence of other pre-existing comorbidities, lower baseline CD4 cell count and unsuppressed viral load levels. In relation to HIV treatment, we analysed the proportions of PLHIV initiated on ART who achieved viral suppression and the duration on ART and found that the proportion of PLHIV on ART decreased with increasing duration on ART and of PLHIV who ever achieved viral suppression, the majority had been on ART for ≤ 3 years. We strongly believe this study would be suitable for describing incident HIV among older people in South Africa as you have suggested.

## 1.9. Although there might be no word limit, the conclusion is quite long and the discussion can be a bit more concise.

**Response:** Thank you for your comment on the length of the conclusion and the discussion. We have revised the conclusion for brevity.
