# Supplementary material for: Kidney function in healthcare clients in Khayelitsha, South Africa: Routine laboratory testing and results reflect distinct healthcare experiences by age for healthcare clients with and without HIV
Source: PLOS Glob Public Health. 2024 May 16;4(5):e0002526. doi: 10.1371/journal.pgph.0002526 (PMC11098392; doi:10.1371/journal.pgph.0002526)
Supplement: Attachment — Submitted filename: Osei_Yeboah_et_al_responsetoreviewer.docx [file pgph.0002526.s006.docx]

**2 March 2024**

**Re: PGPH-D-23-01846R1 Kidney function in healthcare clients in Khayelitsha, South Africa: Routine laboratory testing and results reflect distinct healthcare experiences by age for healthcare clients with and without HIV.**

**Response to Reviewer**

1. *However, there is still some divergence between the conclusion at the end of the article and that presented in the abstract. I kindly request a closer alignment between the conclusions in the article and the abstract for consistency.*

Thank you for your helpful comment requesting a closer alignment between the abstract conclusion section and the manuscript conclusion section. We have implemented this request in full by rearranging the content of the Abstract Conclusion to exactly reflect the messaging in the main text of the manuscript.
